# Research on Medical Security System Based on Zero Trust

**DOI:** 10.3390/s23073774

**Published:** 2023-04-06

**Authors:** Zhiqiang Wang, Xinyue Yu, Peiyang Xue, Yunhan Qu, Lei Ju

**Affiliations:** Beijing Electronic Science and Technology Institute, Beijing 100070, China

**Keywords:** network security threats, zero-trust security system, trust assessment, dynamic access control

## Abstract

With the rapid development of Internet of Things technology, cloud computing, and big data, the combination of medical systems and information technology has become increasingly close. However, the emergence of intelligent medical systems has brought a series of network security threats and hidden dangers, including data leakage and remote attacks, which can directly threaten patients’ lives. To ensure the security of medical information systems and expand the application of zero trust in the medical field, we combined the medical system with the zero-trust security system to propose a zero-trust medical security system. In addition, in its dynamic access control module, based on the RBAC model and the calculation of user behavior risk value and trust, an access control model based on subject behavior evaluation under zero-trust conditions (ABEAC) was designed to improve the security of medical equipment and data. Finally, the feasibility of the system is verified through a simulation experiment.

## 1. Introduction

Due to the impact of the new pneumonia epidemic, more and more countries are paying more attention to the construction of medical system informatization. The computerization of medical systems has therefore become an inevitable trend in the development of international medical services. In this direction, we strongly support the “Internet + Medical Health [1]” work. However, with the increasing informatization of the medical system, the medical system will also face more cybersecurity threats. The medical system contains all kinds of sensitive personal information, such as the age, name, and physical condition of all patients. Once attacked, the medical system may temporarily stop working, affecting the life safety of patients. Therefore, the security design of the medical system is of great importance to the informatization of the medical system.

As one of the emerging network security technologies, zero trust focuses on the protection of information and requires all access subjects to be authenticated and authorized to gain access. In the increasingly complex network environment, it can better resist system attacks. However, zero trust mainly applies to remote office equipment, and the scope of zero-trust research at home and abroad is one-sided. At the same time, a lot of research is being conducted on the combination of medical equipment and the Internet of Things, but more research needs to be carried out on the network security system of medical equipment. Therefore, in order to reduce the network security risks in the intelligent medical system [2], we combine the medical system with the zero-trust security system and propose a medical security system based on zero trust. In addition, based on the RBAC model and the calculation of access subjects’ behavior risk value and trust value, an access control model based on subject behavior evaluation under zero-trust conditions (ABEAC) was designed. The system can better resist attacks on the system, reduce the possibility of medical data leakage, and improve the overall network security of the medical system.

Notations and abbreviations used in this paper are collated in Table 1.

## 2. Related Work

### 2.1. Research Background

Regarding zero trust, a blockchain-based information-sharing solution in a zero-trust environment [3] supports filtering forged information through smart contracts, effective voting, and consensus mechanisms, which can prevent unauthenticated participants from sharing information. A zero-trust protection method is based on network micro-segmentation, security gateway, and device environment awareness [4], which adopts the zero-trust principle and takes user rights as the center to achieve secure application-level access. A dynamic access control strategy based on the zero-trust optical verification network model [5] combines the segmentation method with the zero-trust model so that the system can obtain the ability to control access to valuable assets of the organization or industry. The security protection framework of electric power mobile Internet service based on zero trust [6] makes a collaborative analysis of the key points of security protection based on zero trust from multiple perspectives, such as users, terminals, and applications, and designs multiple dimensions of identity security authentication, continuous trust evaluation, and fine-grained access control, which provides theoretical guidance for the security protection of electric power mobile service. Based on the abstract model of zero-trust architecture and information security technology, a prototype [7] was implemented for the IOS terminal to access enterprise resources in remote office mode securely.

In the medical field, contourlet transform coefficients are used to select the contour region framework of medical images [8], steganography and encryption technologies are combined to protect the confidential data of patients, and medical images are allowed to hide the personal information of patients and other medical data generated by them to ensure the combination of medical images and additional information. A 6G-assisted smart medical environment and centralized user-controlled Single Sign-on (CL-UCSSO) [9] designed a protocol with fast authentication, allowed patients and providers to establish secure communication effectively, and realized a convenient and cost-saving communication multi-server system construction, providing more functions and lower costs. The joint binarized neural network model can derive a reliable medical system [10] if data cannot be shared between medical equipment. It is a system model that simply considers restricted IoT medical equipment. An edge-cloud-based distributed security architecture supporting blockchain [11] can reduce the IoT network edge layer and enhance the capabilities of cloud computing in the healthcare field by detecting security attacks at the cloud layer.

In summary, the zero-trust security model is widely used in network security, and there needs to be more research on medical device network security systems in the medical field.

### 2.2. Technical Background

#### 2.2.1. Zero-Trust Security Model

The zero-trust security model uses policy information management to manage data [12]. This model solves the network security problems caused by excessive reliance on trust and trusted systems. It does not trust any person, device, or system; any access needs to be authorized, and the accuracy of the identity needs to be constantly verified. It further ensures data security, and its core is dynamic trusted access control based on identity.

**Figure 1:**
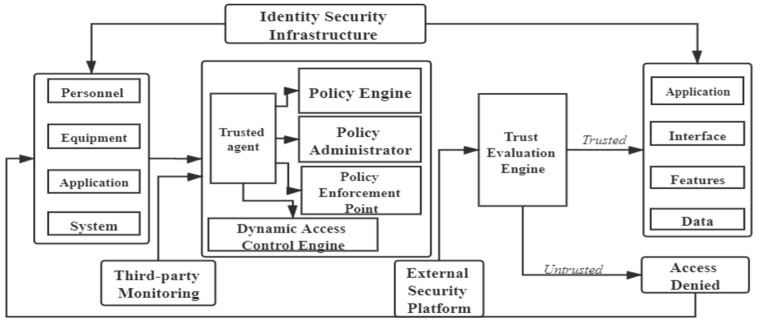
Zero-trust security model architecture components.

The architectural components of the zero-trust security model [13] are shown in Figure 1, which mainly include the trusted agent, the dynamic access control engine, the trust evaluation engine, and the identity security infrastructure.

After the access request, the trusted agent uses the dynamic access control engine to authenticate the identity of the access subject and dynamically decides whether the access subject can access and what operation it can perform if it can access. The dynamic access control engine evaluates the access permissions of all access subjects. Permission evaluation has developed from static rules to dynamic ways based on context attributes and security policies [12]. The trusted agent continuously sends the received information to the trust evaluation engine and combines it with the information in the identity database. Then, it constantly verifies the identity of the access subject by the permission database, the technology of big data, and artificial intelligence. The access subject’s credit level is dynamically evaluated by continuously evaluating the access subject’s credit risk. Finally, the dynamic access control engine generates and maintains the trusted database. Identity management manages the access object’s identity authentication and identity life cycle. Privilege management manages and tracks the authorization policy of the access object.

The zero-trust model undermines boundary control. It assumes that all system parts are untrustworthy, the network is always insecure, trusted areas do not exist, and all devices and users must be authenticated and authorized before access. Compared with the traditional boundary-trust security model, the zero-trust model of any part inside or outside the system is unreliable. Authorization must be verified before access, and each access requires dynamic verification, which can better resist the system’s attacks.

#### 2.2.2. Access Control Model

Dynamic access control is the core part of a zero-trust security system. The commonly used access control models mainly include the discretionary access control model (DAC), the mandatory access control model (MAC), and the role-based access control (RBAC).

Discretionary access control [14] means one or more subjects have an object, and it can allow other subjects to use the object or revoke authorization. This control mode is based on the subject identification and access policy, which specifies tasks the subject can and cannot complete. This method widely applies in both operating systems and database systems.

Mandatory access control [15] is a label-based system mandatory access control mechanism that only applies access control to trusted access subjects, which can ensure that specific access tasks are performed on the system. While mandatory access control management provides the control needed to ensure access to the system safely, it is too restrictive for dynamic environments.

The most important feature of the role-based access control model [16] is the optimization of the authorization management mechanism. This method logically separates users from permissions and introduces roles. In the RBAC model, roles are related to users and permissions, and users match their corresponding roles through roles. Users can match their access rights according to their roles. At the same time, the system can assign different permissions to different users. The RBAC model has been widely used in enterprises, and many scholars have extended and perfected the RBAC model, such as the typical RBAC96 model and RBAC2001 model.

#### 2.2.3. TMBRE Model

Value risk and trust are essential factors affecting system security decisions. However, most existing trust theories ignore the relationship between the value at risk and the trust degree. They do not consider the impact of the value at risk on the system’s security, so it has a certain effect on the correctness of its evaluation. The TMBRE model [17] comprehensively analyzes the relationship between risk value and trust degree, uses the security risk assessment theory to calculate the risk value of the visitor’s behavior, and calculates the corresponding value of trust degree according to the calculation result of risk value. The calculation formula is as follows:

R(e)={(1)μ×Rold(e)(2)δ×Rold(e)+(1−δ)×∑i=0n∑j=0mε×TS(si,tj)×AV(si)×V(si)         

In Equations (1) and (2), $R_{old}\left(e\right)$ represents the current behavioral danger value of an entity, μ represents the risk reduction coefficient, δ represents the degree of optimism about this behavior, ε represents the risk correction factor, $TS\left(s_{i},t_{j}\right)$ represents the threat coefficient of an access event j issued by the access subject to asset i, $AV\left(s_{i}\right)$ represents the defect value of the asset, and $V\left(s_{i}\right)$ represents the value of the asset.

The formula for computing the trust value is as follows:

T(e)={(3)λ×Told(e)+(1−λ)×(θ−R(e)),R∈[θ,10]                   (4)Told(e)+ρ×(θ−R(e)),R∈[0,θ)

In Equations (3) and (4), $T_{old}\left(e\right)$ is the last recorded trust of the entity, λ and ρ represent a trust modifier to ensure that the trust change is within a reasonable range, and θ represents a risk constant to adjust the change in the trust risk value under various circumstances.

## 3. Medical Equipment Cyberspace Security System Based on Zero Trust

To solve the many network security problems in current medical equipment and strengthen the research on the medical device network system, we designed a medical equipment cyberspace security system based on zero trust according to the medical network security specification. In particular, based on the RBAC model, combined with user behavior risk value and trust, we designed an access control model based on subject behavior evaluation under the zero-trust condition (ABEAC), which can better resist attacks on the system, reduce the possibility of medical data leakage, and improve the overall network security of the medical system.

### 3.1. System Construction

The zero-trust-based medical security system is based on the zero-trust principle. No one can be trusted directly (digital identity management for doctors, patients, equipment, development and maintenance, and applications). Anyone needs to be authorized before accessing. The trust level is based on the visitor, the authenticated visitor’s trust level is evaluated dynamically and in real time, and the trust value is automatically adjusted.

**Figure 2:**
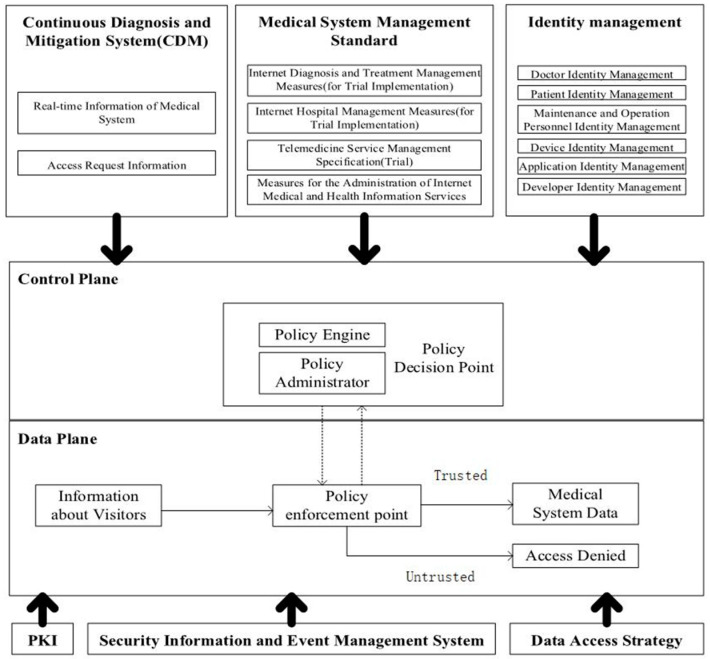
Medical equipment security system based on zero trust.

In this article, a zero-trust-based healthcare security framework is designed, as shown in Figure 2, which specifically includes two parts, the control plane and data plane, where the control plane includes the policy engine and policy administrator and the data plane includes three parts: information about visitors, policy enforcement point, and final decision. Based on the core components of the zero-trust system [18], this framework adds several modules, such as CDM (continuous diagnosis and mitigation system), medical system management standards, identity management, PKI, security information and incident management system, and data access policy.

A continuous diagnosis and mitigation system (CDM) collects current information about medical equipment, including real-time information on the medical system and access request information. It updates the configuration and software organization, such as whether it is running an appropriate patched operating system, the integrity of the medical equipment network software, or whether any known vulnerabilities have appeared in unapproved components and databases.

Medical system management standards are various management systems that must be followed to ensure the normal operation of the medical system, such as Internet diagnostic methods (trial implementation), Internet hospital management methods (trial implementation), telemedicine service management specifications (trial implementation), and Internet medical and health information service management methods.

Identity authentication is a necessary part of the system. The principle of zero trust means that no person, equipment, or system is trusted under any circumstances. Therefore, the medical equipment network security system must first use the trust mechanism based on identity authentication. In the system, all access subjects have a database of corresponding identities, including doctors, patients, devices, applications, developers, and maintenance personnel. After the access subject sends an access request to the medical system, it needs to verify the identity of the access subject and prepare for the later dynamic access evaluation.

The PKI part is responsible for generating and recording the tokens issued by the medical equipment system to resources, services, and applications.

The security information and event management system continuously collects and analyzes security-centric data, then uses data refinement strategies to issue warnings against malicious attacks.

The data access policy is the attributes, rules, and strategies for accessing the medical device database. They are based on the identity and operation of the access subject and can be encoded through the management interface or dynamically generated by the policy engine. They provide basic access rights to accounts and applications/services in the medical device network and are the starting point for authorized resource access.

The policy engine (PE) is responsible for finally determining whether to authorize the access subject and whether the access subject can access the resource. It corresponds to each of the policy manager components. Specifically, based on the use policy of medical equipment and the access operation of medical equipment, the policy administrator (PA) completes the decision of granting, refusing, or revoking the access right of the access subject.

The policy administrator (PA) completes the communication with the policy enforcement point (PEP) by creating a communication path through the control plane. According to the relevant policy execution instructions, the policy administrator decides to open or close the communication channel of the access subject and the access resource. The access subject will produce a specific identity authentication token or credential in applying for medical resources. If the access subject is authenticated, the policy administrator (PA) signals the policy enforcement point (PEP) to open the path between the access subject and the access resource. Otherwise, the policy administrator (PA) signals the policy enforcement point (PEP) to close the connection.

The policy enforcement point (PEP) is a single logical component in the system responsible for enabling, monitoring, and eventually terminating the connection between the medical device and the user based on policy updates sent by the policy administrator (PA). The policy enforcement point (PEP) divides into two different components: the user side and the resource side. According to the dynamic access control policy, it decides the communication connection between the access subject and the access resource.

### 3.2. Dynamic Authentication Process

**Figure 3:**
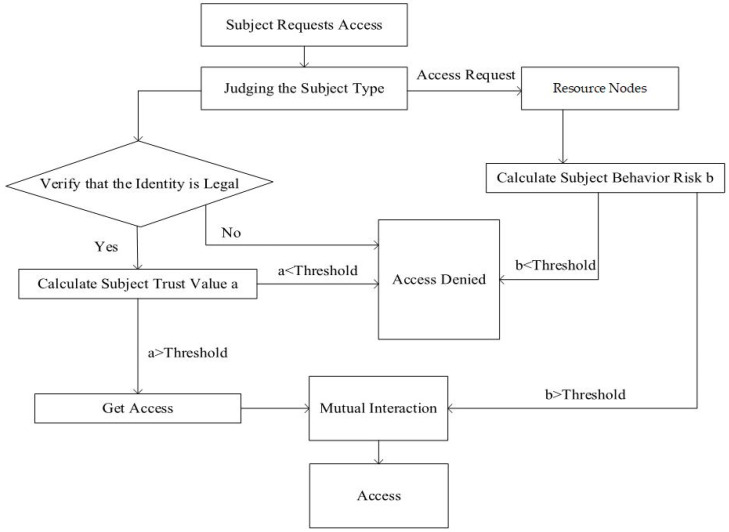
Flow chart of dynamic access control.

In the dynamic access control policy, based on the RBAC model and the calculation results of user behavior risk value and trust degree, we designed an access control model based on subject behavior evaluation under the zero-trust condition (ABEAC). ABEAC is the core part of the zero-trust-based medical security system. In the access control, the access subject is divided into the user and the medical device. The access rights of the access subject are dynamically adjusted by verifying the identity and evaluating the trust degree and behavior risk value, respectively. This not only improves the success rate of interaction between access subject nodes and the resource nodes but also reduces the risk of the illegal operation of legal users and improves the security of medical equipment and data. The dynamic access control flow chart is shown in Figure 3.

Step 1: The access subject submits the access request and judges the subject type. After obtaining the subject type of the user or medical device, it needs to verify the legitimacy of the identity. If it is legal, proceed to the next step; if it is not legal, deny access.

Step 2: Call the trust database according to the access subject type and calculate the subject’s trust value a. If a > threshold, access permission is obtained, and if a < threshold, access is denied.

Step 3: Identify access subject requests and screen resource nodes.

Step 4: Calculate the access subject behavior risk value b. If b > threshold, the access subject can obtain access right; if b < threshold, the access is denied.

Step 5: When the requirements both of a > threshold and b > threshold are met, the two parties can interact to access resources.

### 3.3. Trust Calculation

To provide reliable services and establish a safe medical equipment system, this article combines user behavior risk assessment and identity trust assessment to evaluate user trust. According to the definition of GB/T 20984–2007 Specification for Information Security Risk Assessment of Information Security Technology and related medical equipment standards, through the analysis of information security risk factors in the information system, the user trust and behavior risk values are designed.

#### 3.3.1. Calculation of User Behavior Risk Value

Equation (5) represents the process of risk attenuation when the user has normal behavior, that is, the calculation of the risk value in legitimate access [19], where α ∈ [0.5, 1] is the high-risk attenuation factor, which regulates the attenuation rate of the user’s risk value. Equation (6) calculates the risk value when the user has a threatening behavior, where μ ∈ [1,2] represents the adjustment factor used to adjust the user’s behavior risk under different behavior frequencies. It is the calculation result of the user’s latest behavior risk value; *t* represents the number of user threat behaviors, *CV* is the value of medical data, *V* is vulnerability, and *TA* is threat behavior. According to the information security risk calculation formula and GB/T 20984–2007 Information Security Technology Information Security Risk Assessment Specification, the following definitions are made:

R={(5)α×RV0(6)RV0+μ×t×CV×V×TARV+V+TA                              

*CV* (medical data value): In a cloud environment, the degree of harm caused by a threat varies depending on the importance of different components or resources. Therefore, it is necessary to divide the value of medical data and assign values to different levels of resource values. The higher the level, the more important the resource and the greater its value. The *CV* level of the medical data value is *CV* = {1 *CV* (generally important), 2 *CV* (important), 3 *CV* (more important), 4 *CV* (very important), 5 *CV* (very important)}.

*V* (vulnerability): Vulnerability refers to some weak parts in the cloud, such as software vulnerabilities, backdoors, or hardware system failures. Similar to the classification of data value, the vulnerability of medical data is also classified and assigned. The higher the level, the more serious the vulnerability of medical data and the greater the loss caused by threatened use. *V* = {1 *V* (generally severe), 2 *V* (serious), 3 *V* (more severe), 4 *V* (very severe), 5 *V* (very severe)}.

*TA* (threat behavior): Since any attack is manifested through behavior, this article regards users’ abnormal behavior, breach of contract, and malicious security behavior as threats. Threat behaviors are divided into levels and assigned values according to their impact on resources. The higher the level, the greater the value assigned, indicating that the user behavior has a more serious impact on medical data. The level of threat behavior *TA* is *TA*= {1 *TA* (ignorable impact), 2 *TA* (small impact), 3 *TA* (normally severe impact), 4 *TA* (severe impact), 5 *TA* (very severe impact)}.

#### 3.3.2. User Trust Calculation Formula

Equation (7) is in the high-risk state, where λ∈[0.5, 1] is the trust correction factor in the high-risk state, which adjusts the rate of user trust decline. Equation (8) is in the low-risk state, where ρ∈[0, 0.5] is the trust correction factor in the low-trust risk state to adjust the growth rate of user trust. In the two formulas, R is the user’s risk trust value, t is the number of threat behaviors, T_0_ is the user trust calculated last time, and θ is the user behavior risk threshold constant [20].

T={(7)λt×T0+(1−λ)t×(R−θ),R∈[θ,10]                       (8)T0+ρt×(θ−R),R∈[0,θ)

## 4. Simulation

In the cloud computing environment of medical equipment, dynamic access control can effectively control the illegal behavior of users and protect medical data resources. In order to verify the rationality and effectiveness of this model, this chapter simulates this model from the user behavior evaluation algorithm.

### 4.1. Experimental Data

This article analyzes the calculated behavioral risk values and user trust of two cloud system users when accessing and operating cloud resources ten times, under two situations: 

(1) The change in the risk value and trust level of the user’s normal operation for a long time after a single threat behavior. The specific situation is that when user u1 accessed the system software assigned a value of 6 for the third time, he performed a malicious security behavior assigned a value of 7; the vulnerable value was assigned a value of 5, and the operation was legal at other times.

(2) Changes in user behavior risk and trust under multiple threats. The specific situation is that when the user u_2 accesses the shared data assigned a value of 4 for the second time, he has performed a violation of the assignment value of 5, and the fragile value is assigned a value of 6. The eighth visit to the application software assigned a value of 3 performed an abnormal behavior assigned a value of 2; the fragile value was assigned a value of 2, and the other time operations were legal.

Cloud user assessment needs to be divided into vulnerability levels, medical data value levels, and threat behavior levels. According to the GB/T 20984–2007 Information Security Technology Information Security Risk Assessment Specification and the UBADAC model [20], the classification of vulnerability level is shown in Table 2, the value levels of medical data are classified as shown in Table 3, and the classification of threat behavior is shown in Table 4.

According to the basic resource types of cloud computing, the value levels of medical data are classified as shown in Table 3.

According to the behavior of visiting users, the threat behavior of users is divided into abnormal behavior, breach of contract, and malicious security behavior. The classification of threat behavior is shown in Table 4.

At the same time, in order to have reasonableness and credibility of the experimental data, the initial values of the relevant parameters are set: R_0 (u_1) = 4, R_0 (u_2) = 5, T_0 (u_1) = 5, T_0 (u_2) = 5, α = 0.75, λ = 0.8, ρ = 0.2, θ = 3, μ_1 = 1.4, and μ_2 = 1.8

### 4.2. Analysis of the Relationship between User Behavior Risk and User Trust

In order to improve the rationality of the behavioral risk value and trust degree under the user’s multiple threat behaviors, this article improves the user behavior evaluation algorithm in the TMBRE model. Hereinafter, the TMBRE model is called Model 1, and the design model in this article is Model 2.

**Figure 4:**
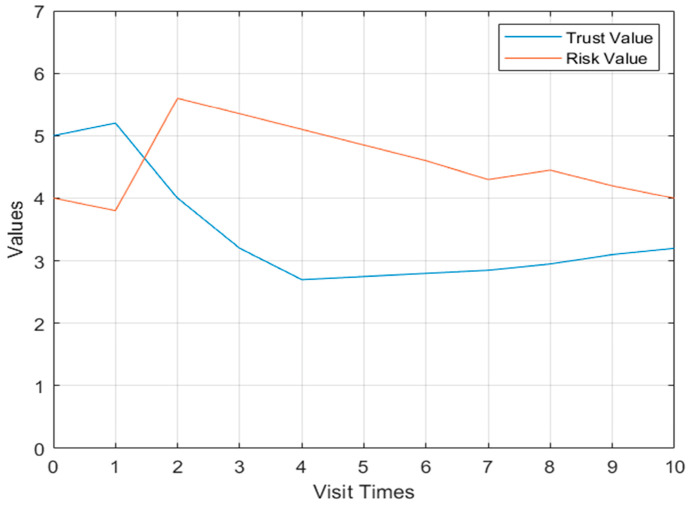
Flow chart of dynamic access control.

For the first case, as shown in Figure 4, when the user has a threat behavior on the second visit, the risk value increases and the trust value decreases in Model 1, but when there is a threat behavior on the eighth visit, the risk value only slightly increases and there is no obvious change.

**Figure 5:**
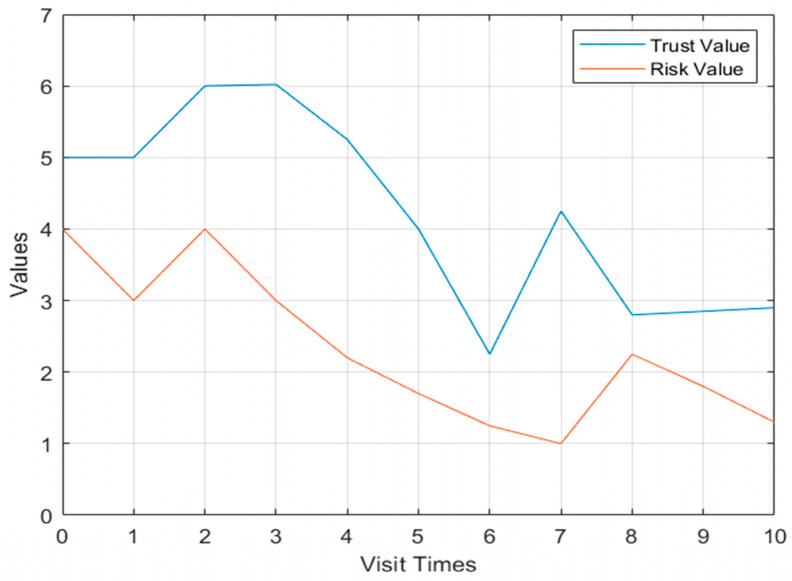
The u_2 behavior risk value and trust change curve Model 2.

In Model 2 in the first case, the change curve of user u_2’s behavioral risk value and trust degree is shown in Figure 5. The user has threatening behavior during the second visit, and the behavioral risk value has the process of increasing. The second threatening behavior appears on the eighth visit, and the behavioral risk value has a sharp increase process, and the degree of trust declines accordingly.

### 4.3. Analysis of the Relationship between Multi-User Behavior Risk and Trust

In the second situation, we conduct a simulation experiment of multi-user behavior risk value and trust degree change and further compare Model 1 and Model 2.

As follows, the user u_1 accessed value level of medical data was shared data assigned 4, breach of contract was 5, and the vulnerability level assignment was 6 for the second time; for the eighth time, the value level of medical data was application software assigned 3, abnormal behavior was 2, and the vulnerability level assignment was 2. Other than that, it was all legitimate access. The user u_2 accessed value level of medical data was systems software assigned 6, abnormal behavior was 2, and the vulnerability level assignment was 4 for the third time; for the seventh time, the value level of medical data was application software assigned 2, breach of contract was 5, and the vulnerability level assignment was 3. Other than that, it was all legitimate access. The user u_3 accessed value level of medical data was shared data assigned 4, abnormal behavior was 3, and the vulnerability level assignment was 2 for the first time; for the fifth time, the value level of medical data was shared data assigned 5, breach of contract was 4, and the vulnerability level assignment was 4; for the seventh time, the value level of medical data was system software assigned 6, abnormal behavior was 2, and the vulnerability level assignment was 8. Other than that, it was all legitimate access. The user u_4 accessed value level of medical data was system software assigned 7, breach of contract was 4, and the vulnerability level assignment was 4 for the fifth time. Other than that, it was all legitimate access.

**Figure 6:**
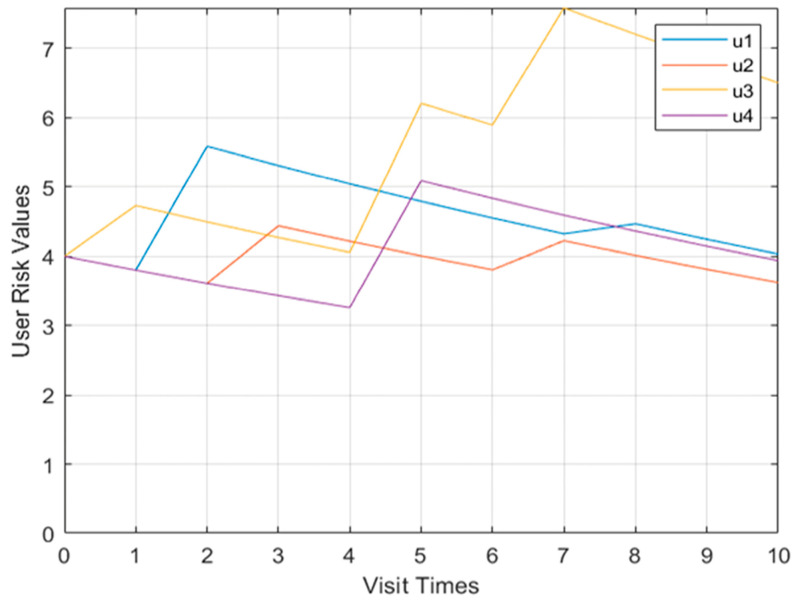
Model multi-user behavior risk value change curve.

Shown in Figure 6 is the change curve of the Model 1 multi-user behavior risk value. The behavioral risk value of the four users in the first few violations of regulations has an obvious trend of increase, but with the next few violations of regulations, the behavioral risk value only increased slightly, and the change was not obvious.

**Figure 7:**
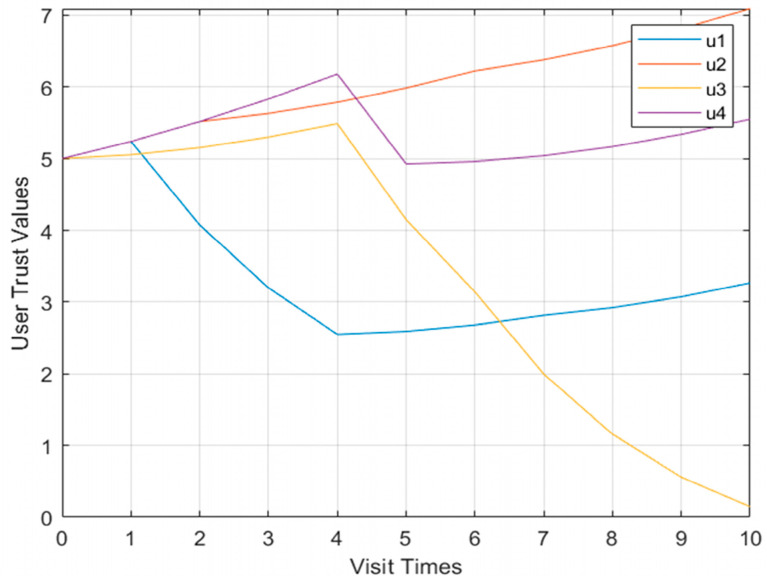
Model multi-user trust value change curve.

Shown in Figure 7 is the curve graph of the Model 1 multi-user user trust degree. When four users perform operations in violation of regulations, the user trust value does not change significantly.

**Figure 8:**
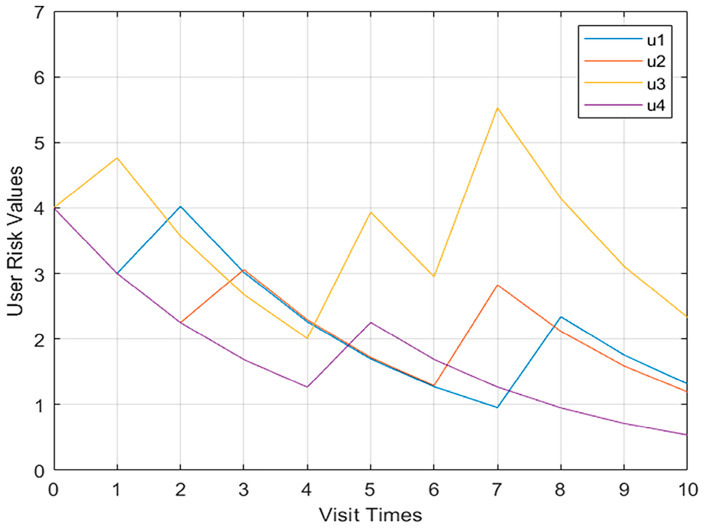
Change curve of multi-user behavior risk value in Model 2.

**Figure 9:**
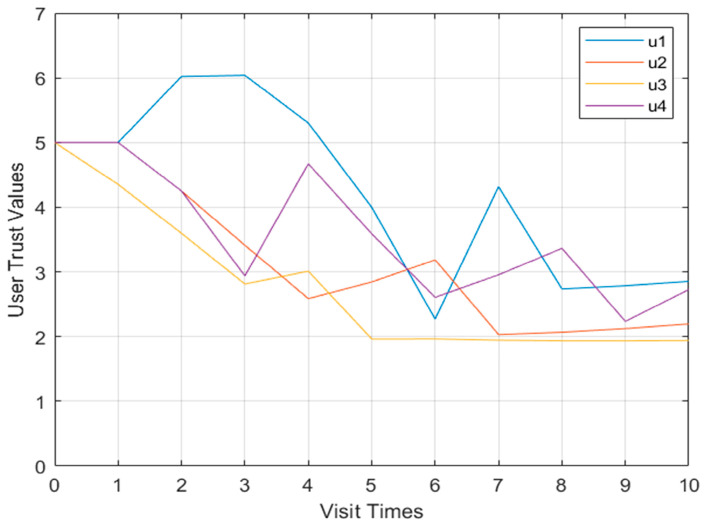
Multi-user trust value change curve of Model 2.

Shown in Figure 8 and Figure 9, respectively, are more than two user models and the behavioral risk value change curve of user confidence. When the four users performed operations in violation of regulations, the behavioral risk values all had a significant upward trend, and the corresponding user trust level also had a corresponding downward trend.

According to the experimental results, the TMBRE model does not consider the impact of the number of threat behaviors on the trust of entities when evaluating trust. However, the ABAEC model makes up for the defect. The trust value of the ABAEC model decreases when the risk value of user behavior increases. When there is only one threat behavior, ABAEC has a sharp increase in the behavior risk value when there is a secondary threat, and the trust degree decreases, which is more in line with the actual situation. When multiple users carry out threatening behaviors, the behavior risk value of the ABAEC model has an obvious upward trend, and the corresponding user trust degree also has a corresponding downward trend, which is more in line with the basic law. The effect is better than the TMBRE model. Therefore, the ABAEC model is more reasonable and effective, and can better protect medical equipment resources.

## 5. Conclusions

With the wide application of Internet technology in the medical field, the intelligent medical system has many network security problems. The problems such as data leakage caused by attacks will pose a serious threat to the life safety of patients. The data security of medical equipment has become one of the important factors affecting the promotion and application of intelligent medical systems. Therefore, it is necessary to strengthen the information security protection of medical equipment and medical information. We designed a zero-trust medical security system based on previous studies to promote medical equipment application and prevent information leakage. In the dynamic access control module, based on the RBAC model and the calculation of user behavior risk value and trust, we designed an access control model based on subject behavior evaluation under zero-trust conditions (ABEAC). We compared it with the TMBRE model in multiple dimensions. Finally, the comparison shows that the ABEAC model is more consistent with the basic law of the changing relationship between behavior risk value and trust. The effect is better than the TMBRE model. The ABEAC model emphasizes dynamic access control, which improves the dynamics and timeliness of the model to ensure the security of medical equipment and data.

At present, the zero-trust medical security system proposed by us can be applied to the medical field to strengthen the information security protection of medical equipment and medical information. However, it also has the problems of cumbersome authentication and low work efficiency. In the future, we will study and improve the following directions:

(1) With the combination of medical devices and the Internet becoming closer and closer, it is necessary to explore a more comprehensive medical equipment model structure, improve the stability of the trust evaluation model, and further improve the security and applicability of the model in the actual application environment.

(2) The access control strategy mainly calculates user behavior risk value and user trust degree, and when both meet the access requirements, the access subject can access. Although the authentication results are accurate, the process is relatively cumbersome. Next, we will shorten the time of identity authentication and improve work efficiency while ensuring that medical equipment is accessed safely.

## Figures and Tables

**Table 1:** Summary of notations and abbreviations.

Notation	Description
R	The risk value
T	The trust value
$R_{old}\left(e\right)$	The last recorded risk value
$T_{old}\left(e\right)$	The last recorded trust value
μ	The risk reduction factor
ε	The risk correction factor
$TS\left(s_{i},t_{j}\right)$	The threat coefficient
$AV\left(s_{i}\right)$	The defect value of the asset
$V\left(s_{i}\right)$	The value of the asset
λ	The trust correction factor
ρ	The trust correction factor
θ	The user behavior risk threshold constant
α	The high-risk attenuation factor
CV	Medical data value
V	Vulnerability
TA	Threat behavior

**Table 2:** Vulnerability level.

Grade	Assignment	Describe
V	8–9	If used by threats, the damage to assets is very serious.
IV	6–7	If used by threats, serious damage to assets.
III	4–5	If used by threats, the damage to assets is generally serious.
II	2–3	If used by threats, the damage to assets is minimal.
I	0–1	If used by threats, the damage to assets can be ignored.

**Table 3:** Value level of medical data.

Grade	Assignment	Cloud Resources	Describe
V	8–9	Infrastructure	Infrastructure includes server pools, storage pools, etc. In the cloud system, the security attributes of these resources will cause very serious losses if they are destroyed.
IV	6–7	Systems software	System software includes an operating system, database, etc. In a cloud system, damage to the security attributes of these resources will cause more serious losses.
III	4–5	Shared data	Shared data include databases that need to share data. In a cloud system, the security attributes of these resources will cause moderate losses if they are compromised.
II	2–3	Application software	Application software includes common software such as OFFICE. In a cloud system, damage to the security attributes of these resources will cause a certain degree of loss.
I	0–1	Portal resources	Portal resources include daily information such as announcements. In a cloud system, the security attributes of these resources are destroyed, causing very little loss.

**Table 4:** Threat behavior division.

**Grade**	**Assignment**	**Cloud Resources**	**Describe**
III	7–9	Malicious security behavior	Malicious security behaviors include high-frequency applications for resources, attacks through Trojan horses, viruses, etc., which may bring huge security risks to the system and damage the system.
II	4–6	Breach of contract	Violation of agreement refers to the violation of the content of the cloud service level agreement, which may affect the normal operation of the system.
I	0–3	Abnormal behavior	Abnormal behavior includes abnormal operating habits, abnormal operating content, etc. The damage it causes to the system is small or even negligible.

## Data Availability

The data used to support the findings of this study are available from the corresponding author upon request.
